# Identification of novel biomarkers for sepsis diagnosis via serum proteomic analysis using iTRAQ‐2D‐LC‐MS/MS

**DOI:** 10.1002/jcla.24142

**Published:** 2021-11-26

**Authors:** Meng Li, Rongrong Ren, Molei Yan, Shangzhong Chen, Chen Chen, Jing Yan

**Affiliations:** ^1^ Department of Clinical Laboratory Zhejiang Hospital Hangzhou China; ^2^ Department of Intensive Care Zhejiang Hospital Hangzhou China

**Keywords:** biomarkers, Isobaric Tags for Relative and Absolute Quantitation, proteomics, sepsis, serum

## Abstract

**Background:**

Sepsis is a common cause of morbidity and mortality in the ICU patients. Early diagnosis and appropriate patient management is the key to improve the patient survival and to limit disabilities in sepsis patients. This study was aimed to find new diagnostic biomarkers of sepsis.

**Methods:**

In this study, serum proteomic profiles in sepsis patients by iTRAQ2D‐LC‐MS/MS. Thirty seven differentially expressed proteins were identified in patients with sepsis, and six proteins including ApoC3, SERPINA1, VCAM1, B2M, GPX3, and ApoE were selected for further verification by ELISA and immunoturbidimetry in 53 patients of non‐sepsis, 37 patients of sepsis, and 35 patients of septic shock. Descriptive statistics, functional enrichment analysis, and ROC curve analysis were conducted.

**Results:**

The level of ApoC3 was gradually decreased among non‐sepsis, sepsis, and septic shock groups (*p* = 0.049). The levels of VCAM1 (*p* = 0.010), B2M (*p* = 0.004), and ApoE (*p* = 0.039) were showing an increased tread in three groups, with the peak values of B2M and ApoE in the sepsis group. ROC curve analysis for septic diagnosis showed that the areas under ROC curve (AUC) of ApoC3, VCAM1, B2M, and ApoE were 0.625, 0.679, 0.581, and 0.619, respectively, which were lower than that of PCT (AUC 0.717) and CRP (AUC 0.706), but there were no significant differences between each index and PCT or CRP. The combination including four validated indexes and two classical infection indexes for septic diagnosis had the highest AUC‐ROC of 0.772.

**Conclusion:**

Proteins of ApoC3, VCAM1, B2M, and ApoE provide a supplement to classical biomarkers for septic diagnosis.

## Graphical abstract

New diagnostic biomarkers of sepsis were screened by iTRAQ2D‐LC MS/MS, and proteins of ApoC3, VCAM1, B2M, and ApoE provide a supplement to classical biomarkers for septic diagnosis.

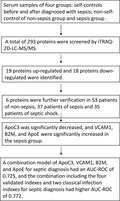

## 1 INTRODUCTION

Sepsis might lead to poor organ function or insufficient blood flow when the body responds to an infection,^1^ which are common causes of morbidity and mortality in the intensive care unit (ICU) patients. It is estimated that there are annually 19.4 million sepsis cases with potentially high deaths in the high‐income countries as North America, Europe, Asia, and Australia. Considering a higher prevalence of sepsis in the low‐ and middle‐income countries,^2^ it is suspected that the global epidemiological burden of sepsis is still difficult to be relieved.

Early diagnosis and appropriate patient management are the key to improve the survival and to limit disabilities in sepsis patients.^3^ Sepsis is a heterogeneous syndrome, as a consequence, it is challenging to identify at early course of the disease, even based on the sepsis 2.0 or sepsis 3.0 diagnostic criteria. Proved by the extensive laboratory and clinical studies on sepsis, biomarkers are able to provide adjunctive information about the pathogenesis of sepsis and guide rapid diagnosis. Procalcitonin (PCT) level rises quickly after the onset of infection, serving as a superior biomarker for evaluating suspected septic patients.^4^ C‐reactive protein (CRP), interleukin‐27 (IL‐27), and neutrophil CD64 (nCD64), with different sensitivity and specificity, were infection markers for early diagnose of sepsis.^5^ Besides serving as infection markers, heparin‐binding protein (HBP) and sphingosine‐1‐phosphate, which could induce endothelial cell dysfunction, were also involved in the pathogenesis and development of sepsis.^6^, ^7^, ^8^ It is worth mentioning that the increase of HBP level can be detected 10.5 h prior to the development of organ dysfunction, which provides a reference for the early diagnosis and clinical management of sepsis.^9^ Host immune system response is invoked early in sepsis to regulate both pro‐inflammatory and anti‐inflammatory responses. HLA‐DR and PDL1 expression on monocytes, TNF production by LPS‐stimulated whole blood cells, were potential biomarkers for innate immunity, and PD1 expression on CD4^+^ or CD8^+^ cells, IFN‐γ production by T cells, and number of circulating regulatory T cells were potential biomarkers for adaptive immunity in sepsis.^10^ In 2010, Pierrakos and Vincent^11^ summarized that at least 178 different sepsis biomarkers have been reported. Only 16 factors were evaluated specifically for the early diagnosis of sepsis, and 5 of these, IL‐12, interferon‐induced protein 10 (IP‐10), Group Ⅱ phospholipase 2 (PLA2‐Ⅱ), CD64, and neutrophil CD11b with high sensitivity and specificity up to 90%. However, only few biomarkers have been used in the clinical testing so far.

As been called “one of the oldest and most elusive syndromes in medicine,”^12^ different biochemical and immunological pathways are involved in sepsis, and a variety levels of proteins have changed in human serum. In this sense, a set of biomarkers works superior to a single biomarker. Isobaric tag for relative and absolute quantification labeling coupled with two‐dimensional liquid chromatography‐tandem mass spectrometry (iTRAQ‐2DLC‐MS/MS) is a proteomics method comparing samples between different groups to screen out a batch of differentially expressed proteins for further identification.^13^ In recent years, several iTRAQ‐2D‐LC‐MS/MS studies on sepsis were performed. Su et al.^14^ brought insights into the prognosis of sepsis using iTRAQ‐2D‐LC‐MS/MS. They identified seven urinary proteins and verified that downregulated LAMP‐1 level may be useful for prognostic assessment of sepsis. Cao et al.^15^ proved that proteins involved in the acute phase response, coagulation signaling, atherosclerosis signaling, lipid metabolism, and production of nitric oxide, and reactive oxygen species were associated with community‐acquired pneumonia which would induce sepsis in the elderly. Jiao et al.^16^ compared serum proteins of septic rats with controls, together with different time points of the survivor and non‐survivor rats, finding five proteins were tightly correlated with the presence of sepsis after verified, and four proteins were related to the prognosis of sepsis. Bian et al.^17^ found the mechanism of H2 in the treatment of sepsis mice by proteomic approaches, which might be helpful for the clinical application of H2 in sepsis patients. The development of experimental techniques and methods provides a technical basis for exploring effective biomarkers for the early diagnosis of sepsis.

In the study, the changes in serum proteome between sepsis and non‐sepsis groups were investigated by iTRAQ‐2D‐LC‐MS/MS, and differentially expressed proteins were quantitative identified and validated in patients with non‐sepsis and sepsis. The candidate protein biomarkers were validated as predictors for the diagnosis of sepsis. Our study provides potential biomarkers for the early diagnosis of sepsis.

## 2 MATERIALS AND METHODS

### 2.1 Patients and control subjects

In order to study the early warning and standardized diagnosis and treatment system of sepsis, "database of the whole process management of early warning and diagnosis and treatment of sepsis," which was jointly developed by Zhejiang Hospital and Zhejiang University, were recorded completely the status and treatment of patients with sepsis before and after diagnosis. Based on this database, adult patients with suspected infection admitted to the department of critical care medicine of Zhejiang Hospital, the Second Affiliated Hospital of Zhejiang University, the First Affiliated Hospital of Sun Yat‐Sen University, West China Hospital of Sichuan University, and Ningbo First Hospital from May 2014 to October 2015 were enrolled.

Adult patients with clinically suspected infection exhibiting one or more of the following indicators were included (1) body temperature of<36°C or >38°C, (2) respiratory rate of >20 breaths/min, (3) pulse rate of>90 beats/min, (4) white blood cell (WBC) count of>12.0 × 10^9^/L or <4.0 × 10^9^/L, or immature granulocyte of >10%, and (5) chief complaint of fever or chills. Cases were excluded if they refused to sign the informed consent, or age<18 years, or diagnosed with malignant tumors. Patients were evaluated four times: at enrollment, the first day, the second day, and the third day according to Sepsis 2.0.^18^ All the “severe sepsis” diagnosed by Sepsis 2.0 were defined as “sepsis” according to Sepsis 3.0,^1^ and others were defined as "non‐sepsis" in this article. Body temperature, pulse and respiratory rates, SOFA score and APACHE score, and blood samples were analyzed at the first evaluation. This study was approved by the Ethics Committee of Zhejiang Hospital (No. 2015‐94K).

The levels of PCT (Roche Diagnostics, USA) and CRP (Beckman coulter, Brea, CA, USA) were measured by Zhejiang Hospital according to the manufacturer's instructions.

### 2.2 Abundant protein depletion and protein digestion

To reduce the influence of individual variation and increase precision and accuracy of the data in the proteomics study,^19^ equal amounts of six different samples were mixed to produce a sample pool, and each sample pool was labeled and detected two times as technical replicates. High‐abundance serum proteins (such as albumin, IgG, IgA, fibrinogen, transferrin, haptoglobin, etc.) were removed from the mixed sample using the Human 14 Multiple Affinity Removal System Columns (Agilent). The low‐abundance proteins were concentrated and determined by the Bradford method.

A total of 300 μg protein was washed with an extraction buffer (8 M Urea, 150mM Tris‐HCl pH8.5), 14,000*g* centrifuged 30 min, repeated three times. And then, each sample was alkylated with 100 μl 50 mM iodoacetamide (IAA), incubated at room time, and away from light for 30 min. The samples were washed with 100 μl UA buffer for three times to remove the exceed IAA and washed with 100 μl 1/10 dissolution buffer (100 mM triethylammonium formate) for three times to provide an alkaline environment of digestion. Finally, each sample was digested by 40 μl Trypsin buffer (2 μg Trypsin in 40 μl 1/10 Dissolution buffer) on constant temperature mixer (eppendorf thermomixer C) for 18 h at 37°C with 300rpm.

### 2.3 iTRAQ labeling and strong cationic exchange fractionation

Digested samples were labeled with an iTRAQ reagent‐8ples Multiplex kit (Applied Biosystems SCIEX) following the manufacturer's protocol. Four groups of mixed serum samples with two technical duplicates were set up: self‐controls before diagnosed with sepsis were labeled iTRAQ reagent 113, 117, and diagnosed sepsis group were labeled 114, 118; non‐self‐control of non‐sepsis group were labeled iTRAQ reagent 115, 119, and sepsis group were labeled 116, 121.

The eight labeled sample groups were mixed and separated using Polysulfoethyl column (4.6 × 100 mm, 5 µm, 200 Å, PolyLC Inc) with strong cation exchange (SCX) fractionation by AKTA Purifier 100 (GE Healthcare). Thirty SCX fractions were collected and combined to six SCX fractions based on SCX chromatograms. Each SCX fraction was dried by centrifugal evaporation (Eppendorf Concentrater plus) and desalted with Empore™ SPE‐C18 Cartridges (Sigma).

### 2.4 LC‐MS/MS analysis

The SCX fractions were analyzed using Easy‐nLC1000HPLC system (Thermo Fisher Scientific) connected to Q‐Exactive mass spectrometer (Thermo Fisher Scientific). Mobile phase A was 0.1% formic acid solution, and mobile phase B was 0.1% formic acid‐acetonitrile solution (contain 84% acetonitrile). The chromatographic column was balanced by 95% Mobile phase A. SCX fractions were loaded by an autosampler onto trapping column (2 cm*100 μm 5 μm‐C18, Thermo scientific EASY column) to enrich and desalt, and then the samples were loaded onto an analytical column (75 μm*100 mm 3 μm‐C18) to separate. The gradient was as follows: 0–105 min, mobile phase B from 0 to 10%; 105–110 min, mobile phase B from 10% to 30%; 110–120 min, mobile phase B maintain in 100%.

The mass spectrometer worked in positive ion mode, and the MS spectrum was obtained in the range of 300–1800 m/z. The MS scan resolution of Q‐Exactive was set to 70,000 and MS/MS scan resolution was 17,500. In the obtained MS spectrum, the top ten most intense signals were selected for further MS/MS analysis. The isolation window was 2 m/z, and ions were fragmented through higher energy collisional dissociation, and the normalized collision energies were 30 eV. The maximum ion implantation time of the measured scan was set at 10 ms, and the maximum ion implantation time of the full scan mode was set at 60 ms. The automatic gain control target value of the full scan mode was set to 3e6. The automatic gain control target value of the MS Magi MS was set to 5e4. The dynamic exclusion time is 40 s.

### 2.5 Data analysis

RAW files were identified, quantified, and analyzed by softwares Maxquant1.4.1.2 and perseus1.4.1.3. This database is used by Uniprot human database (a total of 141,033 protein sequences, with a download date of 2014.12.19). Request search parameters were as follows: two maximum trypsin missed cleavages; peptide mass tolerance ±20 ppm; fragment mass tolerance 0.1 Da; fixed modifications were carbamidomethyl modification (C), iTRAQ‐8plex (N‐terminus), and iTRAQ‐8plex (K); variable modifications were oxidation (M), and result filter parameters were PSM‐level FDR≤0.01 and Protein‐level FDR≤0.01. The ion peak intensity reflected the relative abundance of the peptide and protein, and the quantitative ratio of the peptide segments was normalized against the median ratio value of the internal standard sample. The proteins with an expression ratio between the two groups >1.20 (upregulated proteins) or <0.83 (downregulated proteins) were chosen for further research. We used the "cluster Profiler"^20^ package in R for the Gene Ontology (GO) annotations of the differentially expressed proteins (DEPs).GO database analyzed the cellular component, molecular function (MF), and biological process of proteins (BP) (*p* value cutoff = 0.05, *q* value cutoff = 0.05). The protein‐protein interaction network of these DEPs was constructed by Search Tool for the Retrieval of Interacting Genes database (STRING, https://www.string‐db.org/) and visualized by Cytoscape software. The PPI pairs were extracted with a minimum required interaction score: >0.38. The degree of each protein node was calculated by Cytoscape software.

### 2.6 Differential proteins validation

SerumApoC3, SERPINA1, VCAM1 (Abcam), and GPX3 protein (CUSABIO Biotech) levels were detected using ELISA kits according to the manufacturer's instructions. Briefly, serum samples were diluted with dilution factors of 1:4000, 1:200, 1:200, and 1:2000 for ApoC3, SERPINA1, VCAM1, and GPX3, respectively. If the concentration of SERPINA1 was over 2000 ng/ml or below 31.7 ng/ml, the experiment was repeated with dilution factors of 1:2000 or 1:20. Diluted samples and standards were added to microtiter wells coated with corresponding antibodies. SERPINA1 kits and VCAM1 kits were a one‐step ELISA, with antibody cocktail added together; while ApoC3 kits and GPX3 kits were classical two‐step ELISA, with specific detection antibody and enzyme conjugated second antibody added step by step. Finally, TMB substrate was added, and the reaction was then stopped by the addition of Stop Solution. Absorbance was measured on iMark microplate spectrophotometer (Bio‐Rad, Inc.) at 450 nm. The best‐fit line was determined by regression analysis using a four‐parameter logistic curve‐fit (Microplate 5.0 software, Xinghe Inc.). The sample concentration was determined from the standard curve and multiplied by the dilution factor.

Serum B2M protein and ApoE levels were detected on AU5821 and AU5421 Automatic biochemical analyzer (Beckman coulter). B2M was agglutinated with latex particles coated with B2M antibody, and the turbidity was directly proportional to the concentration of B2M by immunoturbidimetry (AUTEC Diagnostica). ApoE was also detected by immunoturbidimetric method (Saike Biotechnology).

### 2.7 Statistical analysis

Continuous variables were presented as mean ± standard deviation (SD) or the median with interquartile range (IQR), and categorical variables as numbers and percentages. Comparisons among non‐sepsis, sepsis, and septic shock groups were performed using ANOVA test for means, chi‐square test for the numbers, and Kruskal‐Wallis *H* test for medians. Comparisons between non‐sepsis and sepsis groups were performed using *t* test for means, chi‐square test for the numbers, and Mann‐Whitney *U* test for medians. Receiver operating characteristic curves were constructed to show each cut‐off levels and assess the diagnostic validity of ApoC3, B2 M, VCAM1, ApoE, PCT, and CRP alone and in combination. *p* values of <0.05 were regarded as statistically significant. The SPSS software system 22.0 (SPSS), GraphPad Prism 7 (GraphPad Software), and Medcalc 11.4 (MedCalc Software bvba) were used for calculations.

## 3 RESULTS

### 3.1 Characteristics of the patients

A total of 125 patients, containing 53 patients of non‐sepsis, 37 patients of sepsis, and 35 patients of septic shock, were enrolled in the study. The characteristics of the patients were compared in Table 1. Compared to non‐sepsis patients, sepsis patients and septic shock patients presented increased temperature, pulse rate, SOFA score and APACHE score, and decreased mean arterial pressure (*p *< 0.05). Infection marker of PCT was showing increased trend, and CRP was shoving a peak value in sepsis group (*p *< 0.05).

**TABLE 1:** Characteristics of the study population

Characteristics		Non‐sepsis (*n* = 53)	Sepsis (*n* = 37)	Septic shock (*n* = 35)	(*X^2^*/*F*/*Z*)	*p* value
Male, n (%)		44 (83.0)	28 (75.7)	21 (60.0)	5.909	0.052
Age, year (mean ± SD)		65.4 ± 20.3	61.6 ± 15.9	62.1 ± 18.2	0.556	0.575
Vital signs at diagnosis, mean ± SD						
Temperature, °C		37.8 ± 0.9	37.9 ± 1.4	38.4 ± 1.1	3.511	0.033*
Pulse rate, beats/min		96.3 ± 19.8	116.0 ± 14.7	117.4 ± 21.0	17.674	<0.001*
Respiratory rate, breaths/min		20.6 ± 5.6	23.7 ± 6.7	21.2 ± 6.4	2.838	0.062
Mean arterial pressure, mm Hg		74.8 ± 10.6	70.0 ± 8.9	61.5 ± 9.0	19.403	<0.001*
Laboratory findings and Scores, median (25th,75th)						
PCT, μg/L	0.42 (0.12, 1.88)		2.49 (0.60, 7.66)	7.09 (1.57, 15.2)	32.250	<0.001*
CRP, mg/L	76.99 (36.97, 154.91)		166.06 (87.62, 297.33)	154.43 (49.37, 267.19)	11.638	0.003*
APACHE Ⅱscore	16.0 (10.0, 22.0)		20.0 (13.5, 24.5)	20.0 (16.0, 28.0)	11.743	0.003*
SOFA score	7.0 (5.0, 12.0)		9.0 (6.5, 12.5)	15.0 (12.0, 17.0)	33.586	<0.001*

Abbreviations: APACHE Ⅱ, acute physiology and chronic health evaluation Ⅱ; CRP, C‐reactive protein; PCT, procalcitonin; SOFA, sequential organ failure assessment.

*p < 0.05.

### 3.2 Serum proteins identification and relative quantification

**FIGURE 1:**
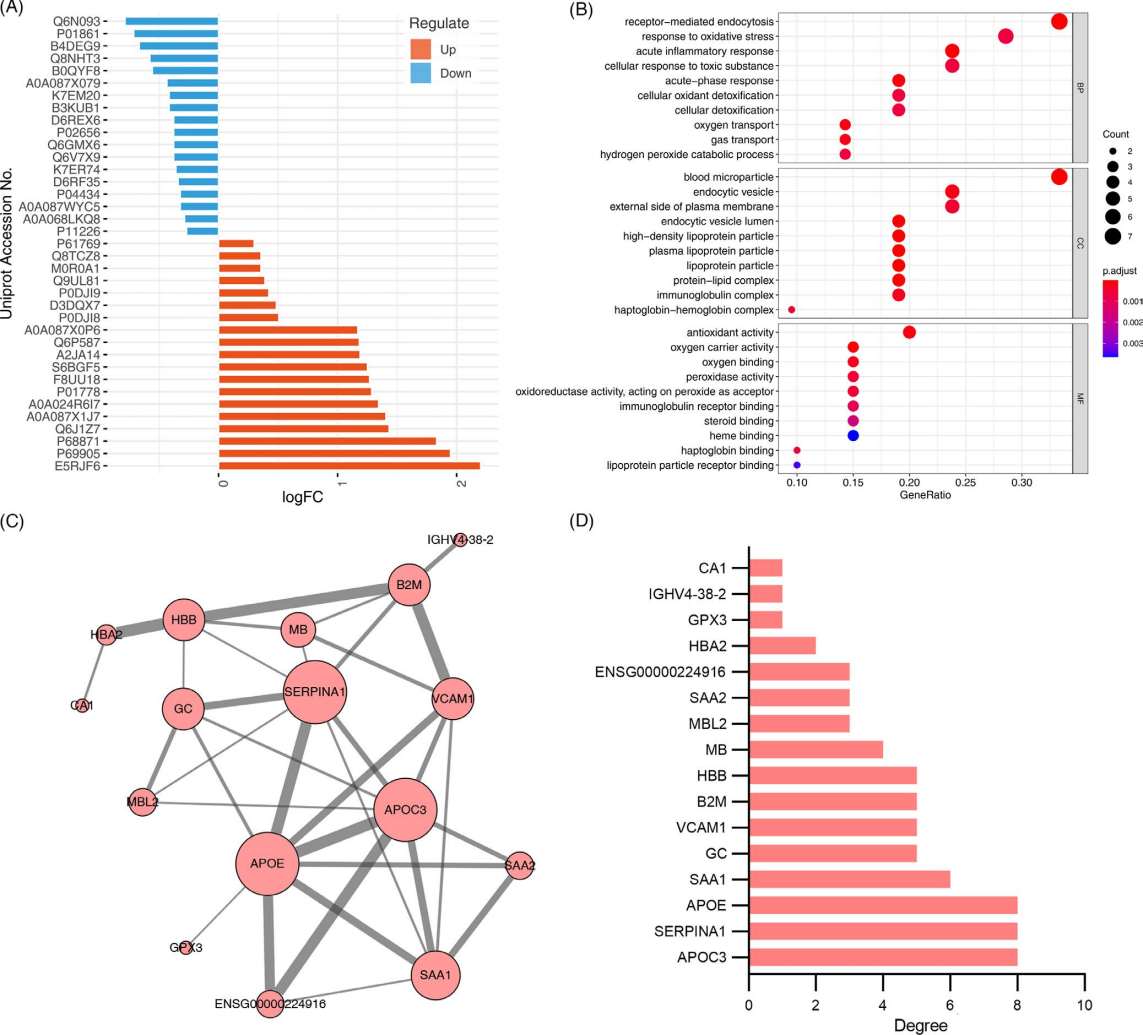
Functional enrichment analysis of the differentially expressed serum proteins in non‐sepsis and sepsis patients. (A) The differentially expressed proteins in non‐sepsis and sepsis patients; (B) significantly enriched GO terms of the differentially expressed proteins;(C) the protein‐protein interaction network constructed with the differentially expressed proteins; (D) the differentially expressed proteins with degree of connectivity

All iTRAQ‐labeled proteins were identified and quantitatively analyzed with 2D‐LC‐MS/MS. A total of 293 proteins were screened in the four groups. Among them, 37 differentially expressed proteins were identified between samples with and without sepsis, of which 19 proteins were upregulated (>1.20‐fold, *p *< 0.05) and 18 proteins were downregulated (< 0.83‐fold, *p*< 0.05) (Figure 1A, Table 2).

**TABLE 2:** Differentially expressed proteins and the ratios of sepsis to non‐sepsis samples quantified by iTRAQ‐ 2DLC‐MS/MS

Protein ID	Gene symbol	Protein Name	Ratios
Decreased protein in sepsis			
Q6N093	DKFZp686I04196	Putative uncharacterized protein DKFZp686I04196 (Fragment)	0.58
P01861	IGHG4	Ig gamma−4 chain C region	0.61
B4DEG9		cDNA FLJ58448, highly similar to Bromodomain‐containing protein 8	0.63
Q8NHT3		Fructose‐bisphosphate aldolase	0.67
B0QYF8	MB	Myoglobin (Fragment)	0.68
A0A087X079	IGHG1	Ig gamma−1 chain C region	0.74
B3KUB1		cDNA FLJ39520 fis, clone PUAEN2001526, highly similar to Inositol polyphosphate multikinase (EC 2.7.1.151)	0.75
K7EM20	YWHAE	14–3–3 protein epsilon (Fragment)	0.75
Q6V7X9		Protein C (Fragment)	0.77
Q6GMX6	IGH@	IGH@ protein	0.77
P02656	APOC3	Apolipoprotein C‐III	0.77
D6REX6	FAM153A	Protein FAM153A (Fragment)	0.77
K7ER74	APOC4‐APOC2	Protein APOC4‐APOC2	0.78
D6RF35	GC	Vitamin D‐binding protein	0.79
A0A087WYC5	IGHG1	Ig gamma−1 chain C region	0.80
P04434		Ig kappa chain V‐III region VH (Fragment)	0.80
A0A068LKQ8		Ig heavy chain variable region (Fragment)	0.82
P11226	MBL2	Mannose‐binding protein C	0.83
Increased protein in sepsis			
E5RJF6	CA1	Carbonic anhydrase 1 (Fragment)	4.60
P69905	HBA2	Hemoglobin subunit alpha	3.86
P68871	HBB	Hemoglobin subunit beta	3.56
Q6J1Z7	HBB	Hemoglobin beta (Fragment)	2.70
A0A087X1J7	GPX3	Glutathione peroxidase 3	2.65
A0A024R6I7	SERPINA1	Serpin peptidase inhibitor, clade A (Alpha−1 antiproteinase, antitrypsin), member 1, isoform CRA_a	2.54
P01778		Ig heavy chain V‐III region ZAP	2.44
F8UU18	VCAM1	Vascular cell adhesion molecule 1 (Fragment)	2.41
S6BGF5		IgG H chain	2.38
A2JA14		Anti‐mucin1 heavy chain variable region (Fragment)	2.28
Q6P587	FAHD1	Acylpyruvase FAHD1, mitochondrial	2.27
A0A087X0P6	IGKV2D−29	Protein IGKV2D−29	2.25
P0DJI8	SAA1	Serum amyloid A−1 protein	1.42
D3DQX7	SAA1	Serum amyloid A protein	1.40
P0DJI9	SAA2	Serum amyloid A−2 protein	1.34
Q9UL81		Myosin‐reactive immunoglobulin light chain variable region (Fragment)	1.31
M0R0A1	RPL37A	60S ribosomal protein L37a	1.28
Q8TCZ8	APOE	Apolipoprotein E (Fragment)	1.28
P61769	B2MG	Beta−2‐microglobulin	1.23

Further, the enriched functional categories by 37 differentially expressed proteins were analyzed. In biological process categories (BP), most of the differentially expressed proteins were involved in receptor‐mediated endocytosis, response to oxidative stress, acute inflammatory response, cellular response to toxic substance, acute‐phase response, detoxification, and transport. In cellular component categories, the differentially expressed proteins were mostly enriched in the extracellular, including blood microparticle, endocytic vesicle, external side of plasma membrane, endocytic vesicle lumen, lipoprotein particle, and protein‐lipid complex. In molecular function categories (MF), these proteins were mainly enriched in oxidative stress and immune response, including antioxidant activity, oxygen carrier activity, oxygen binding, peroxidase activity and oxidoreductase activity, and immunoglobulin receptor binding (Figure 1B). As shown in Figure 1CandD, ApoC3, ApoE, and SERPINA1 were the most outstanding protein with connectivity degree = 8, followed by SAA1 (degree = 6), GC (degree = 5), VCAM1 (degree = 5), B2 M (degree = 5), HBB (degree = 5), MB(degree = 4), MBL2 (degree = 3), SAA2 (degree = 3), ENSG00000224916 (degree = 3), HBA2 (degree = 2), GPX3 (degree = 1), IGHV4‐38–2 (degree = 1), and CA1(degree = 1).

### 3.3 Differential expression proteins validation

Based on the fold changes, the degree of connectivity in PPI network, and appropriateness of the samples, six proteins were selected for further verification in 53 patients of non‐sepsis, 37 patients of sepsis, and 35 patients of septic shock, including serum apolipoproteinC3 (ApoC3), serpin family A member 1 (SERPINA1), vascular cell adhesion molecule 1 (VCAM1), beta‐2‐microglobulin (B2M), glutathione peroxidase 3 (GPX3), and apolipoprotein E (ApoE). As shown in Table 3, the level of ApoC3 was gradually decreased among non‐sepsis, sepsis, and septic shock groups (*p* = 0.049). The levels of VCAM1 (*p* = 0.010), B2M (*p* = 0.004), and ApoE (*p* = 0.039) were showing an increased tread in three groups, and B2M and ApoE were showing peak values in the sepsis group. While the levels of GPX3 (*p* = 0.947), SERPINA1 (*p* = 0.605), and showed no significant difference among the groups (Table 3).

**TABLE 3:** Analysis of serum ApoC3, SERPINA1, VCAM1, B2 M, GPX3, ApoE levels

	Non‐sepsis (*n* = 53)	Sepsis (*n* = 37)	Septic shock (*n* = 35)	*Z*	*p* value
ApoC3 (μg/ml)	85.1 (57.0, 109.2)	67.0 (32.8, 98.1)	62.0 (326.6, 99.0)	6.021	0.049*
SERPINA1 (μg/ml)	177.6 (22.4, 400.0)	211.4 (40.4, 400.0)	102.9 (23.7, 400.0)	1.004	0.605
VCAM1 (ng/ml)	1639.8 (1,274.5, 1,978.5)	1759.0 (1312.1, 2500.9)	2053.4 (1532.0, 2887.2)	9.275	0.010*
B2M (mg/L)	2.57 (1.50, 7.56)	6.36 (2.51, 12.99)	5.06 (2.04, 17.13)	11.14	0.004*
GPX3 (μIU/L)	122.8 (79.9, 196.3)	132.4 (78.2, 235.1)	123.5 (93.4, 191.9)	0.109	0.947
ApoE (mg/L)	42.8 (36.3, 54.2)	48.2 (38.2, 74.1)	41.7 (26.9, 49.7)	6.514	0.039*

* Indicates that the difference is statistically significant after Kruskal‐Wallis *H* test and *p *< 0.05.

### 3.4 Biomarker levels for diagnosis of sepsis

**FIGURE 2:**
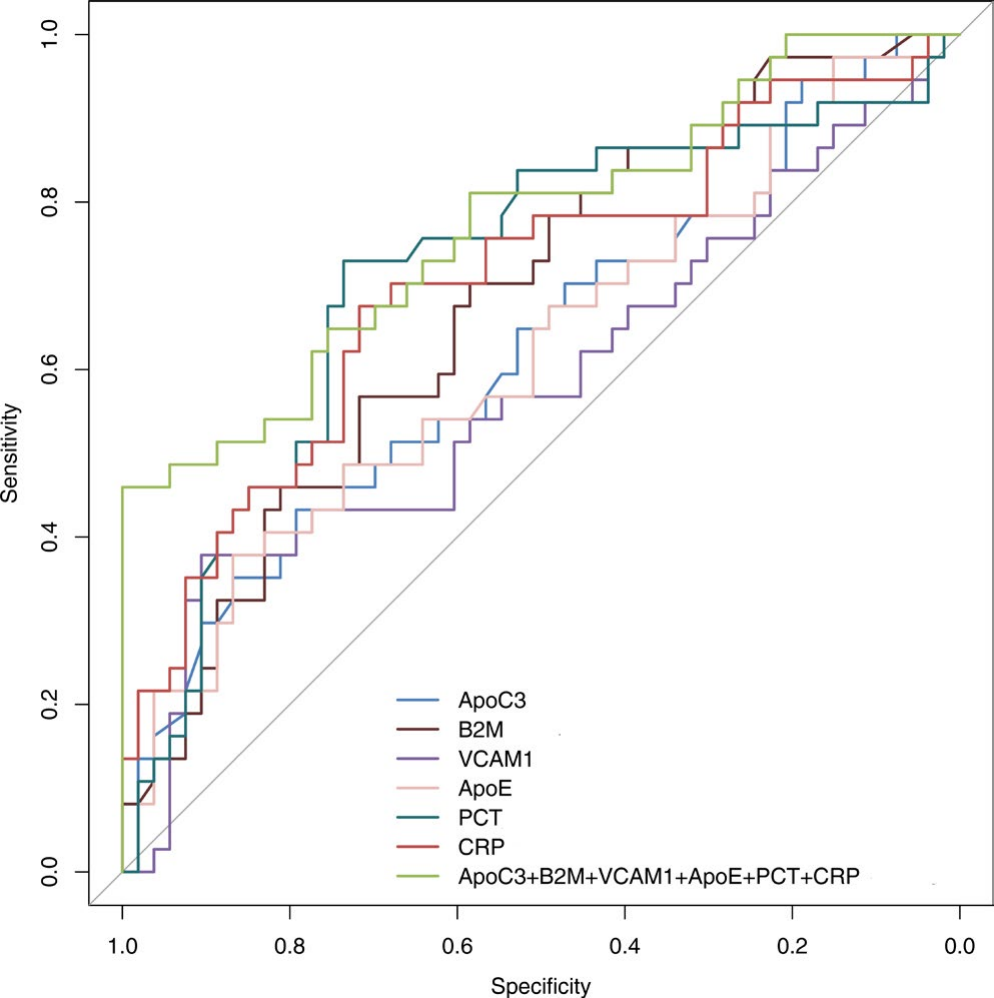
Receiver operating characteristic curves were applied for assessing diagnostic tests

Table 4 shows that comparing with classical infection indexes of PCT and CRP, the validated indexes of ApoC3, VCAM1, and ApoE have higher specificity and positive predictive value, but lower sensitivity and negative predictive value, while B2M has approximate specificity and positive predictive value. To assess the validated indexes’ diagnostic efficiency of sepsis, ROC curve analysis showed that the areas under ROC curve (AUC) of ApoC3, VCAM1, B2M, and ApoE were 0.625 (95%CI: 0.517–0.725), 0.679 (95%CI: 0.573–0.774), 0.581 (95%CI: 0.472–0.684), and 0.619 (0.510–0.719), respectively, and the AUC of PCT and CRP were 0.717 (95%CI: 0.612–0.807) and 0.706 (95%CI: 0.600–0.797), respectively.The AUC of validated indexes were lower than that of PCT and CRP, but there were no significant differences between each index and PCT or CRP (*p *> 0.05). Each validated index respectively combined with classical infection indexes of PCT and CRP, and only the combination of VCAM1 had AUC‐ROC of 0.745, which was better than that of VCAM1 (*p* = 0.018). Further, the combination including four validated indexes and two classical infection indexes for septic diagnosis had the highest AUC‐ROC of 0.772, which was significantly better than that of ApoC3, VCAM1, and ApoE respectively, besides B2M. (Figure 2, Table 5).

**TABLE 4:** Analysis of diagnostic efficacy of various indexes in the diagnosis of sepsis

	Cut‐off	Sensitivity (%)	Specificity (%)	PPV (%)	NPV (%)
ApoC3	≤54.532 μg/ml	43.24 (27.5–60.4)	79.25 (65.5–88.7)	59.26(39.0–77.0)	66.67(53.6–77.7)
B2M	>3.7 mg/L	70.27 (52.8–83.6)	58.49 (44.2–71.6)	54.17(39.3–68.4)	73.81(57.7–85.6)
VCAM1	>2216.8 ng/ml	37.84 (22.9–55.2)	90.57 (78.6–96.5)	73.68(48.6–89.9)	67.61(55.3–78.0)
ApoE	>62.45 mg/L	37.84 (22.9–55.2)	86.79 (74.0–94.1)	66.67(43.1–84.5)	66.67(54.2–77.3)
PCT	>1.04 ng/ml	72.97 (55.6–85.6)	73.58(59.4–84.3)	65.85(49.3–79.4)	79.59(65.2–89.3)
CRP	>115.59 mg/L	67.57 (50.1–81.4)	71.70(57.4–82.8)	62.50(45.8–76.8)	76.00(61.5–86.5)

Abbreviations: NPV, Negative predictive value; PPV, Positive predictive value.

**TABLE 5:** Analysis of diagnostic efficacy of various indexes in the diagnosis of sepsis

	AUC	95% CI	*p* *	*P* #	*P&*	*P@*
ApoC3	0.625	0.517–0.725	0.246	0.292	0.081	0.014
B2M	0.679	0.573–0.774	0.547	0.729	0.296	0.121
VCAM1	0.581	0.472–0.684	0.067	0.161	0.018	0.012
ApoE	0.619	0.510–0.719	0.244	0.3114	0.130	0.028
PCT	0.717	0.612–0.807	–	0.863	–	0.332
CRP	0.706	0.600–0.797	0.863	–	–	0.093
ApoC3 + PCT + CRP	0.730	0.626–0.818	0.829	0.321	–	–
B2M + PCT + CRP	0.738	0.635–0.825	0.683	0.274	–	–
VCAM1 + PCT + CRP	0.745	0.642–0.831	0.607	0.241	–	–
ApoE + PCT + CRP	0.726	0.622–0.815	0.872	0.464	–	–
ApoC3 + B2M + VCAM1 + ApoE	0.725	0.620 −0.814	0.907	0.786	–	–
ApoC3 + B2M + VCAM1 + ApoE+PCT + CRP	0.772	0.671–0.853	0.332	0.093	–	–

* Compared with PCT, # compared with CRP, *&* compared with PCT and CRP, *@* compared with the combination of ApoC3, B2M, VCAM1, ApoE, PCT and CRP.

## 4 DISCUSSION

Sepsis lacks effective early diagnostic biomarkers, which could lead to rapidly disease progresses and poor prognosis. With the development of proteomics, genomics, transcriptomics, and metabolomics, the researchers attempt to set up a batch of biomarkers for the diagnosis and prognosis of sepsis, which provide higher diagnostic specificity and sensitivity than a single biomarker. In this study, 37 differential proteins were screened between sepsis and non‐sepsis by iTRAQ‐2D‐LC‐MS/MS method. Moreover, the significantly enriched GO terms and the PPI network were performed to analyze the differentially expressed proteins. Among the 37 differential proteins screened above, we selected six (VCAM1, ApoC3, B2M, SERPINA1, GPX3, and ApoE) to validate their diagnostic value by quantitative analysis of multiple samples. Out of them, VCAM1, ApoC3, B2M, and ApoE had significant differences, while GPX3 and SERPINA1 did not. The combination of four proteins was exhibited a ROC curve with an AUC of 0.772 in diagnosing sepsis, which was higher than the AUC of PCT (0.717) and CRP (0.706).

Endothelium dysfunction causes impaired perfusion, tissue hypoxia, organ dysfunction, and subsequent sepsis. VCAM1 appears to be associated with sepsis. VCAM was upregulated in sepsis and downregulated in non‐survivors.^21^ Elevated serum level of VCAM1 was a more powerful predictor for septic encephalopathy in adult community‐onset sepsis on admission.^22^ We found that the level of VCAM1 was showing an increased tread among non‐sepsis, sepsis, and septic shock groups, with a peak value in the sepsis group.

Numerous studies demonstrated thatβ2‐microglobulin (B2M) was a biomarker of renal function, whose serum levels were associated with glomerular filtration rate.^23^ The level of serum B2M often in combination with the increase of cystatin C and urea nitrogen were used for evaluating drug, cardiovascular risk, kidney transplantation, and kidney injuries with other etiologies.^24^, ^25^, ^26^ In our study, serum B2M level was showing an increased tread among non‐sepsis, sepsis, and septic shock groups, with a peak value in the sepsis group. B2M is a circulating factor positively associated with the sepsis patients and indicative of a suspected sepsis‐induced kidney injury. Meanwhile, B2M is a component of major histocompatibility complex class 1 (MHC I) molecules, which has no transmembrane region of a ternary membrane protein complex on all nucleated cells. B2M was involved in immune reactions, such as mucosal immunity, tumor surveillance, and immunoglobulin homeostasis.^27^, ^28^ Since the new recognition of the relationship between hyper‐inflammatory response and immune suppression, the immune homeostasis of sepsis attracted a lot of attention.^11^ In the B2M null mice, very limited amounts of MHC class I molecules could be detected on the cell surface, and CD8 T cells could not develop which were a subset of T cells involved in the development of acquired immunity.^29^, ^30^ B2M knockout mice with lethal intra‐abdominal sepsis exhibited decreased systemic inflammation and survived significantly longer than wild‐type mice.^31^ Thus, on one hand, B2M participated in the cell surface expression of MHC class I, contributes to the stability of the peptide‐binding groove and the presenting antigenic peptides to cytotoxic T cells, and on the other hand is not desired that the overreaction of B2M promotes inflammation leading to organ injury.

In our study, serum ApoC3 level was gradually decreased among non‐sepsis, sepsis, and septic shock groups, which was mainly secreted by the liver, as a potential biomarker for the liver pathogenesis.^32^ ApoC3 can be glycosylated with most abundant glycoforms by an O‐linked disaccharide galactose linked to N‐acetylgalactosamine (Gal‐ GalNAc) and less abundant glycoforms by fucosylated glycan moieties.^33^, ^34^ ApoC3 glycol‐isoform ratios were altered in patients with sepsis and other severe systemic diseases, which might be an important indicator for diagnostic, prognostic, and therapeutic status.^35^ In addition, this study showed that the serum level of ApoE was increased in sepsis and septic shock group, with a peak value in the sepsis group. Previous studies had shown that ApoE genotype was associated with sepsis. Wild‐type (ε3) was associated with decreased incidence of sepsis,^36^ and disease‐associated (ε4) alleles were also found increased in coagulation system failure in human sepsis, which was a determinant of the human innate immune response to multiple TLR ligands.^37^ Changes of lipid metabolism in sepsis were also reported in other apolipoproteins. ApoA and ApoB concentrations in sepsis had a negative correlation with procalcitonin (PCT),^38^ the plasma concentrations of ApoM were dramatically decreased in sepsis patients, which were contributed to the increased vascular leakage observed in sepsis.^39^ Whether apolipoproteins can be used as a panel of biomarkers for diagnose and predict sepsis remains to be further studied.

SERPINA1, also known as alpha‐1 antitrypsin, is a serine protease inhibitor, which can inhibit a variety of serine endopeptidases. GPX‐3 is a key selenoprotein with antioxidant properties,^40^ which is abundant in serum and plasma. Although there was no statistical difference in the quantitative detection of SERPINA1 and GPX‐3 in this study, previous studies had shown positive outcome. SERPINA was found association with sepsis in urinary proteomics^41^ and genomics,^42^ and C‐terminal alpha‐1 antitrypsin peptide might be a promising discriminatory biomarker for sepsis with immunomodulatory functions.^43^ Sepsis‐related decline of GPX‐3 protein concentration resulted in the decrease in GPX‐3 bioactivity.^44^ Extensive experiments are needed to verify the clinical application of SERPINA1 and GPX‐3 in sepsis and uncover the mechanisms of them as a biomarker for sepsis.

Infection markers such as PCT and CRP are commonly used for the adjuvant diagnosis of sepsis,^4^, ^5^ and their role in the diagnosis and prediction of sepsis is still being explored.^45^, ^46^ In this study, a diagnostic model by a combination of the four validated indexes and two infection markers was established, with the highest AUC‐ROC of 0.772 than that of PCT or CRP, although there were no significant differences between each combination AUC and AUC of PCT or CRP. B2M, VCAM1, and ApoE, besides ApoC3, already can be detected in clinical laboratory, which provided the clinical application possibility for septic diagnosis.

## 5 CONCLUSIONS

In this study, the combination of ApoC3, VCAM1, B2M, and ApoE proteins were screened and identified as biomarkers for sepsis by using iTRAQ‐2D‐LC‐MS/MS method. This is a new combination and a supplement to classical biomarkers such as procalcitonin or C‐reactive protein.

## CONFLICT OF INTEREST

No conflict of interest exists in the submission of this article.

## AUTHOR CONTRIBUTIONS

Jing YAN conceived the study, reviewed the draft, and commented on it; Mo‐lei YAN and Shang‐zhong CHEN enrolled patient and established the database; Meng LI and Chen CHEN conducted the experiments; Meng LI and Rong‐rong REN analyzed the data and wrote the draft. All authors reviewed the article and approved the final article.

## Data Availability

The authors certify that this article reports original clinical trial data. The datasets used and/or analyzed during the current study are available from the corresponding author on reasonable request.
